# Nmnat mitigates sensory dysfunction in a *Drosophila* model of paclitaxel-induced peripheral neuropathy

**DOI:** 10.1242/dmm.032938

**Published:** 2018-06-12

**Authors:** Jennifer M. Brazill, Beverley Cruz, Yi Zhu, R. Grace Zhai

**Affiliations:** 1 Department of Molecular and Cellular Pharmacology, University of Miami Miller School of Medicine, Miami, FL 33136, USA; 2 School of Pharmacy, Key Laboratory of Molecular Pharmacology and Drug Evaluation (Yantai University), Ministry of Education, Collaborative Innovation Center of Advanced Drug Delivery System and Biotech Drugs in Universities of Shandong, Yantai University, Yantai, Shandong 264005, China

**Keywords:** Paclitaxel, Chemotherapy, Neuropathy, Microtubules, Nmnat, Neuroprotection

## Abstract

Chemotherapy-induced peripheral neuropathy (CIPN) is the major dose-limiting side effect of many commonly used chemotherapeutic agents, including paclitaxel. Currently, there are no neuroprotective or effective symptomatic treatments for CIPN. Lack of understanding of the *in vivo* mechanisms of CIPN has greatly impeded the identification of therapeutic targets. Here, we optimized a model of paclitaxel-induced peripheral neuropathy using *Drosophila* larvae that recapitulates aspects of chemotherapy-induced sensory dysfunction*.* We showed that nociceptive sensitivity is associated with disrupted organization of microtubule-associated MAP1B/Futsch and aberrant stabilization of peripheral sensory dendrites. These findings establish a robust and amenable model for studying peripheral mechanisms of CIPN. Using this model, we uncovered a critical role for nicotinamide mononucleotide adenylyltransferase (Nmnat) in maintaining the integrity and function of peripheral sensory neurons and uncovered Nmnat's therapeutic potential against diverse sensory symptoms of CIPN.

## Abstract (precis)

**Summary:** Neurotoxic side effects of chemotherapy are poorly understood. Here, the authors optimize a *Drosophila* model of paclitaxel-induced sensory dysfunction, which is then used to explore the neuroprotective capacity of Nmnat.

## INTRODUCTION

Chemotherapy-induced peripheral neuropathy (CIPN) is a serious neurotoxic side effect of standard chemotherapy regimens. Over 68% of patients experience neuropathic symptoms after chemotherapy (Seretny et al., 2014), which mostly present as bilateral sensory symptoms including numbness; paresthesia such as burning, tingling and sharp pain; allodynia and hyperalgesia; and sensory ataxia. These distressing side effects can lead to the delay or discontinuation of treatment, threatening oncological outcomes and prognosis. While symptoms may resolve post-chemotherapy, up to 30% of patients develop chronic sensory dysfunction that can persist for months or even years, negatively impacting the quality of life for survivors (Seretny et al., 2014). The current paucity of treatments for CIPN presents a critical need to develop effective neuroprotective strategies (Hershman et al., 2014).

Paclitaxel is a microtubule-targeting agent used in chemotherapy, either alone or in conjunction with other drugs, against breast, ovarian and lung cancer, among other solid tumors. Paclitaxel binds along the microtubule lattice and suppresses microtubule dynamics, leading to cell cycle arrest and tumor cell death (Jordan et al., 1993). As neurons rely heavily on microtubules for their complex structural and functional integrity, disrupted microtubule dynamics are also a putative source of the neurotoxic side effects of paclitaxel. One of the prevailing hypotheses of paclitaxel-induced peripheral neuropathy assumes that disrupted microtubule-based axonal transport results in a dying-back degeneration of peripheral sensory neurons (Persohn et al., 2005; Tanner et al., 1998). However, newer *in vivo* models using more clinically relevant chemotherapeutic doses suggest that axon degeneration is not prevalent (Bennett et al., 2011), and evidence of microtubule disruption is primarily derived from cell culture studies, which do not account for the complexity of the injury pathway *in vivo* (Duggett et al., 2016). As such, new mechanistic insights into the pathophysiology of paclitaxel-induced peripheral neuropathy, indeed of all neurotoxic chemotherapy regimens, are urgently needed.

A major challenge in understanding CIPN is the difficulty of characterizing the cellular and physiological sequelae *in vivo* using traditional rodent models. *Drosophila* and mammalian peripheral sensory neurons share key architectural properties and a similar anatomical arrangement: finely branched free nerve endings innervate the epidermis, cell bodies are housed in the periphery, and long axons project from the periphery to synapse in the central nervous system (Im and Galko, 2012). These common features are particularly important in the study of CIPN, as each neuronal compartment differentially contributes to the selective vulnerability of peripheral sensory neurons to chemotoxic side effects (Fukuda et al., 2017). Further empowering the use of a *Drosophila* larval model of CIPN are the conserved genes and molecular pathways of pain neurobiology (Honjo et al., 2016; Neely et al., 2010; Tracey et al., 2003), which more recently have been extended to encompass mechanisms of sensitization following tissue damage (Babcock et al., 2009; Im et al., 2015). *Drosophila* larvae are an amenable and accessible *in vivo* model suited for probing mechanisms of CIPN at the level of peripheral sensory neurons within the context of a complex circuit.

Here, we present an improved model of CIPN and sensory dysfunction induced by the microtubule-stabilizing agent paclitaxel in *Drosophila* larvae, demonstrating the utility of this powerful model organism for probing the cellular and molecular basis of CIPN. Using this model we uncovered characteristic dose-dependent changes in sensory dendrites of class IV dendritic arborization (C4da) nociceptors under paclitaxel treatment. We identified nicotinamide mononucleotide adenylyltransferase (Nmnat), a neuronal maintenance factor previously well studied for its ability to delay axon degeneration (Coleman and Freeman, 2010), as a critical determinant for C4da neuron-mediated nociceptive sensitivity. Importantly, Nmnat overexpression mitigated paclitaxel-induced hypersensitivity, revealing its therapeutic potential in CIPN.

## RESULTS

### *Drosophila* larvae exposed to paclitaxel treatment experience thermal hypersensitivity

**Fig. 1.**
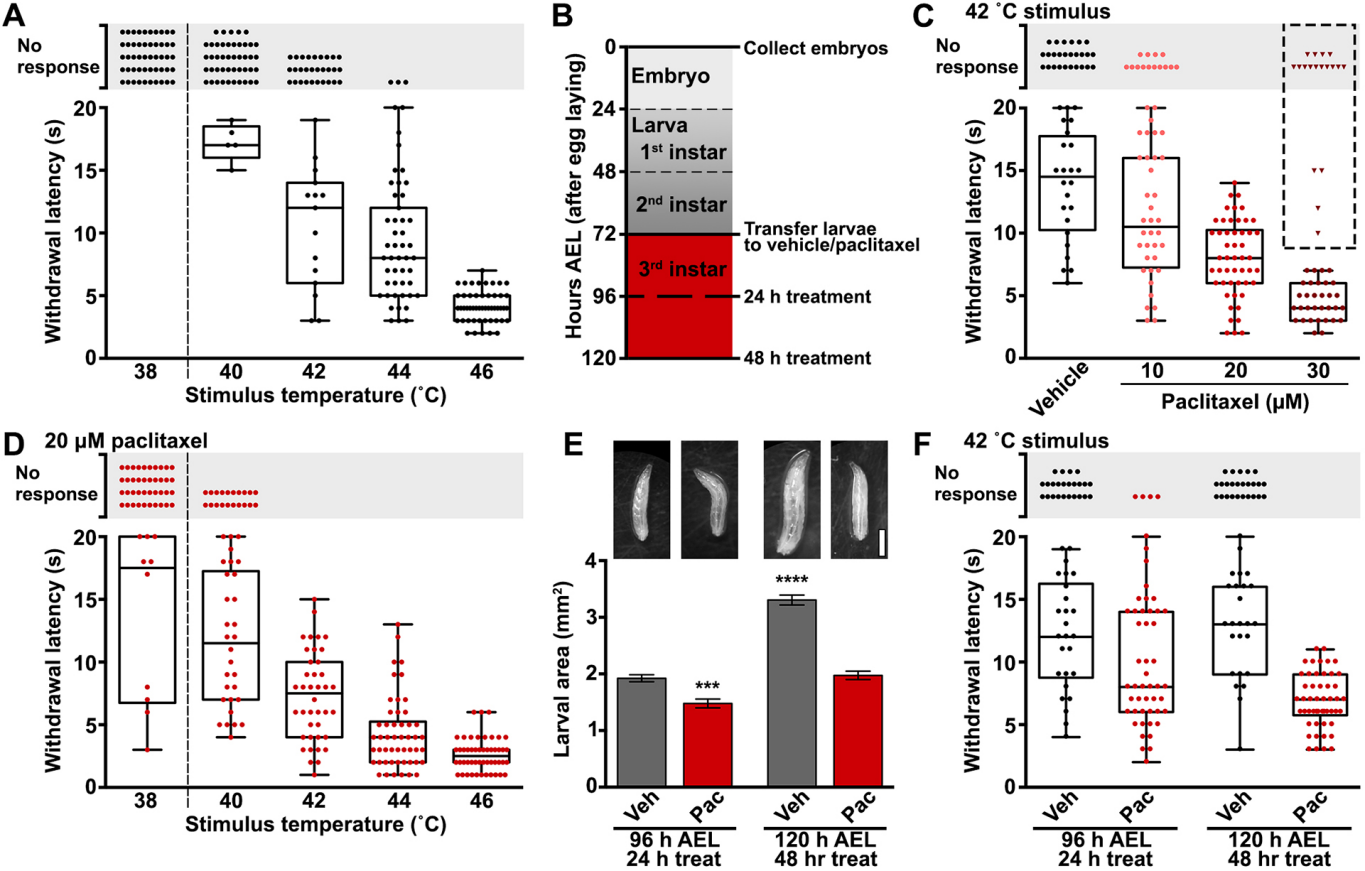
**Novel model of paclitaxel-induced sensory dysfunction using *Drosophila* larvae.** (A) Thermal nociceptive profile of third instar larvae (120 h AEL) stimulated at the indicated temperatures. Each data point represents an individual larva scored for withdrawal latency (box and whiskers) or categorized as no response if aversive withdrawal was not initiated within 20 s of stimulation. No larvae responded at 38°C, indicating sub-threshold stimulation (dashed line). Larvae were transferred to vehicle-laced food at 72 h AEL. *n*=50 larvae tested at each temperature. (B) Paclitaxel treatment paradigm outlining the time course of treatment during larval development. Larvae were age matched during embryo collection, transferred to treatment at early third instar (72 h AEL) and treated for either 24 h or 48 h before testing for thermal nociception or neuropathy at 96 h AEL or 120 h AEL, respectively, during late third instar. (C) Withdrawal response from 42°C stimulation of late third instar (120 h AEL) larvae after 48 h exposure to either vehicle (0.2% DMSO) or 10 µM, 20 µM or 30 µM paclitaxel. Each data point represents an individual larva. Upon 48 h exposure to 30 µM paclitaxel, several larvae exhibited motor disturbances (triangles) and were excluded from calculation of the withdrawal latency (box and whisker). A concentration of 20 µM was selected as being optimal for engendering thermal nociceptive hypersensitivity and is used in the remainder of this study. *n*=50 larvae tested for each treatment condition. (D) Thermal nociceptive profile of 120 h AEL larvae subjected to 48 h paclitaxel treatment stimulated at the indicated temperatures. Paclitaxel-induced hypersensitivity is robustly detected at 42°C stimulation by both an increased response rate of the larvae tested and a decrease in the withdrawal latency of responders compared to vehicle-treated controls (panel A) and is used in the remainder of this study. *n*=50 larvae tested at each temperature in conjunction with vehicle-treated controls in A. (E) Representative images of larvae at age 96 h AEL and 120 h AEL treated with either vehicle (Veh) or 20 µM paclitaxel (Pac) for 24 h or 48 h, respectively, acquired from a dissection microscope fitted with a smartphone adapter. Scale bar: 1 mm. Larval area is presented in mm^2^ and is derived from the length multiplied by the width of each larva. Mean±s.e.m.; one-way ANOVA with Tukey's multiple comparisons test. (F) Thermal nociceptive withdrawal behavior upon 42°C stimulation tested over the time course of treatment. The withdrawal behavior is maintained from 96 h AEL to 120 h AEL during larval development of vehicle-treated controls. Paclitaxel-induces nociceptive hypersensitivity that progresses with the duration of treatment from 24 h and 48 h treatment. *n*=50 larvae at each time point and treatment. ***P<0.001, ****P<0.0001.

*Drosophila* larvae stimulated with gentle touch at a sub-threshold temperature continue peristaltic crawling, whereas larvae presented with noxious thermal stimulation display a corkscrew-like rolling withdrawal (Tracey et al., 2003). This stereotypical behavior has been used as the basis for modeling thermal nociception. We custom-built a digital thermal controller comprising a fine-tip probe that can stimulate a small lateral abdominal area of a *Drosophila* larva at a user-defined temperature based on a previously reported design (Babcock et al., 2009). We tested temperatures spanning the larval nociceptive thermal range on control third-instar larvae [120 h AEL (after egg laying)]. We recorded time to initiate the aversive withdrawal response or classified larvae as non-responders if they did not respond aversely within 20 s of stimulation. Based on this response profile, we defined 38°C stimulation as sub-threshold, as no larvae exhibited aversive rolling behavior at this temperature (Fig. 1A). Upon stimulation from 40 to 46°C, we observed a response profile with an increased proportion of larvae withdrawing, with decreased latency as temperature increased (Fig. 1A). We reasoned that 42°C stimulation afforded the most dynamic range for assessing changes in thermal nociceptive sensitivity, whether manifested as sensory deficits or hypersensitivity upon paclitaxel treatment.

We modified a paclitaxel feeding regimen that has been previously reported to cause overt peripheral neurodegeneration in larvae (Bhattacharya et al., 2012) with the goal of lessening the severity of the neuronal injury and overall drug toxicity. The severity and frequency of CIPN has been shown to correlate with both the dose and duration of paclitaxel treatment (Lee and Swain, 2006). As such, we modified paclitaxel concentration and treatment time to achieve a paradigm suitable for monitoring development of sensory dysfunction in *Drosophila* larvae. As outlined in Fig. 1B, we limited exposure to 48 h spanning the third larval instar (72-120 h AEL). We performed a dose-response analysis of paclitaxel concentration on nociceptive function. Paclitaxel treatment with 10, 20 or 30 µM led to thermal hypersensitivity that increased in severity in a concentration-dependent manner (Fig. 1C). Specifically, larvae treated with paclitaxel were more likely to respond to 42°C stimulation and responded with a decreased latency compared to vehicle-treated larvae (Fig. 1C). Of note, after 48 h treatment with 30 µM paclitaxel, several larvae exhibited locomotor deficits, including lack of coordination, weak crawling ability, inability to roll and immobility (Fig. 1C, triangles). Therefore, we selected 48 h treatment with 20 µM paclitaxel for our treatment paradigm; this schedule produced hypersensitivity without affecting the motor system, consistent with typical patient presentations and rodent models of CIPN. We comprehensively analyzed the sensory dysfunction induced by our paclitaxel treatment paradigm across the nociceptive thermal range (Fig. 1D, compare to vehicle-treated controls in Fig. 1A). Paclitaxel treatment produced both allodynia, responsiveness to sub-threshold stimulation at 38°C, and hyperalgesia, enhanced responsiveness to noxious stimulation (40-46°C). Treatment-induced nociceptive behavioral responses, in terms of larval response rate and mean withdrawal latency, were most readily detected upon 42°C stimulation.

We explored the development of paclitaxel-induced sensory dysfunction over the time course of our treatment paradigm. At 72 h AEL, larval size prohibited reliable behavioral response in the thermal nociception assay, as larvae were often unable to move away from the probe. Larvae grew rapidly and, by 96 h AEL after 24 h of treatment, vehicle-treated larvae reached an average area of 1.9 mm^2^, whereas paclitaxel slightly impeded larval growth to 1.5 mm^2^ (Fig. 1E). At this stage, larval size was compatible with reliable sensory testing and the response rate of the paclitaxel-treated group to 42°C stimulation was significantly elevated compared to vehicle-treated controls, while the mean withdrawal latency was modestly reduced (response rate, 24 h vehicle=52%, 24 h paclitaxel=92%; mean withdrawal latency±s.e.m., 24 h vehicle=12.1±0.9 s, 24 h paclitaxel=9.5±0.7 s; Fig. 1F). Larvae continued to grow through the 120 h AEL stage, although 48 h paclitaxel-treated larvae only reached an average area of 2.0 mm^2^ compared to 3.3 mm^2^ for vehicle-treated controls (Fig. 1E). Importantly, paclitaxel-induced hypersensitivity progressed over the 48 h treatment, whereas vehicle-treated larvae maintained their nociceptive response over this time period (response rate, 48 h vehicle=50%, 48 h paclitaxel=92%; mean withdrawal latency±s.e.m., 48 h vehicle=12.9±0.8 s, 48 h paclitaxel=6.8±0.3 s; Fig. 1F). These data demonstrate that, within the 48 h treatment period spanning developmental stages from 72 h to 120 h AEL, thermal hypersensitivity is a direct consequence of paclitaxel treatment, rather than merely a side effect of restricted larval size. Our results demonstrate that *Drosophila* larvae are susceptible to paclitaxel-induced thermal hypersensitivity and are a robust model for quantitatively studying paclitaxel-induced sensory dysfunction.

### Hypersensitivity-inducing paclitaxel regimen leads to increased density of peripheral sensory dendrites

**Fig. 2.**
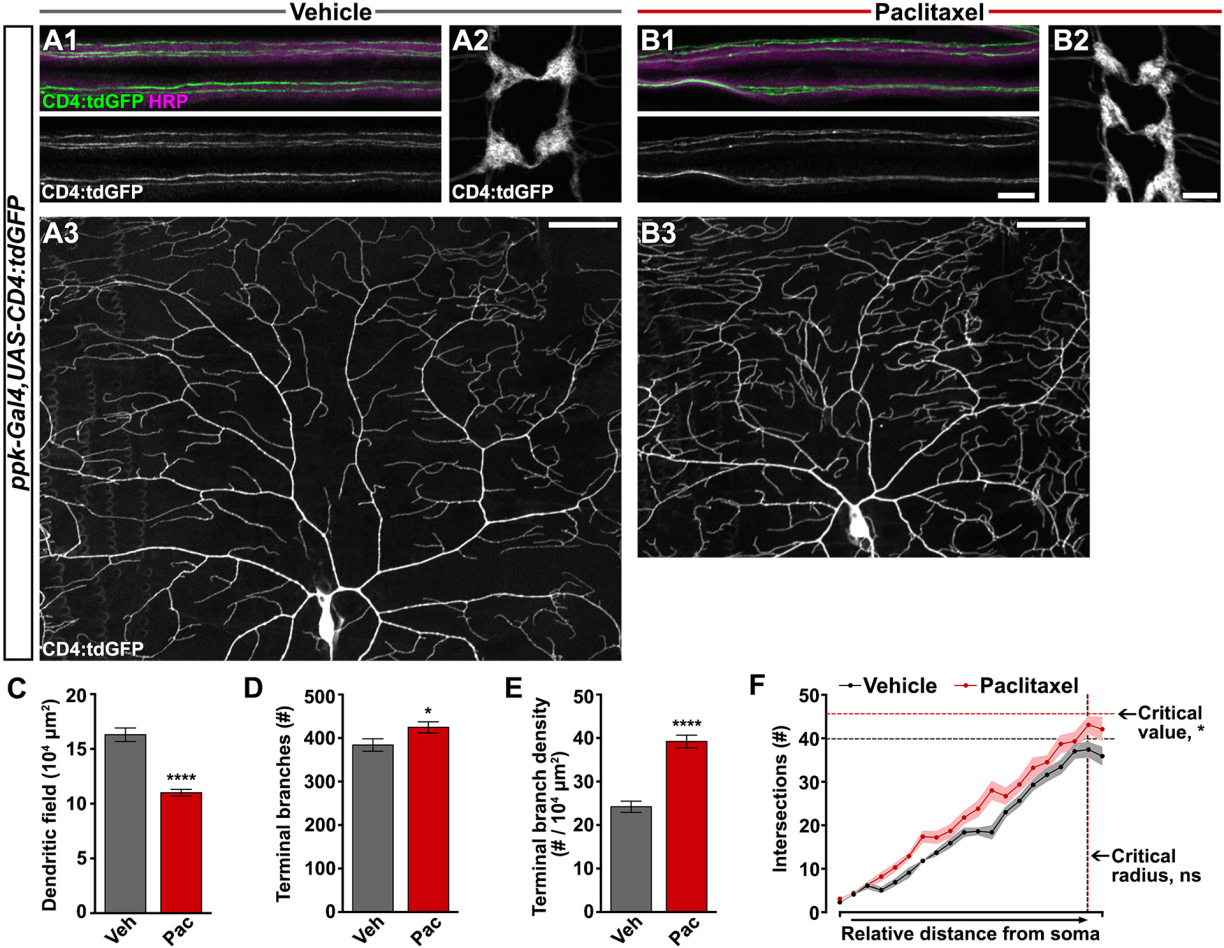
**Paclitaxel treatment increases peripheral sensory dendrite branch density.** (A1-B3) Confocal projections of fixed, filet-dissected vehicle- or paclitaxel-treated larvae showing class IV dendritic arborization (C4da) neuronal compartments labeled by CD4:tdGFP. (1) Individual C4da sensory axons are visible within the HRP-labeled peripheral nerve bundles. (2) C4da axon terminals project to form a ladder-like pattern in the VNC. (3) Dorsal dendrite projections of C4da ddaC shown here with cell body centered near the bottom of the frame. Axon and VNC scale bar: 10 µm; dendrite scale bar: 50 µm. (C-E) Dorsal projections of C4da dendrites labeled by CD4:tdGFP after treatment with either vehicle or paclitaxel were quantified: (C) area of dendritic field measured in ImageJ; (D) number of terminal branches counted manually; and (E) terminal branch density. Mean±s.e.m.; *n*=10 neurons from >5 larvae for each treatment, Student's *t*-test. (F) Sholl profile was performed on dorsal projections of C4da dendrites in ImageJ and plotted as the number of branch intersections with relative distance from the cell body to the anterior/posterior edges of the dendritic field. Solid lines connect mean intersections at each relative shell and shaded regions represent s.e.m.; *n*=10 neurons from >5 larvae for each treatment; interaction: ns, relative distance: *P*<0.0001, treatment group: *P*<0.0001, two-way ANOVA; critical value: *P*<0.05, critical radius: ns, Student's *t*-test. *P<0.05, ****P<0.0001.

We next set out to assess the effects of paclitaxel on the larval class IV dendritic arborization (C4da) neurons, the peripheral polymodal nociceptors responsible for thermal nociception (Hwang et al., 2007). C4da neurons project elaborate dendritic arbors that innervate the epidermis. These neurons conduct nociceptive signals through long axons to synapse with central neurons in the VNC (ventral nerve cord). To facilitate a detailed and comprehensive analysis of nociceptor morphology after paclitaxel treatment, we utilized the Gal4/upstream activator sequence (UAS) binary expression system to drive the CD4:tdGFP membrane-targeted reporter specifically within C4da neurons (*ppk-Gal4*, *UAS-CD4:tdGFP*). We chose this reporter because it evenly distributes along the neuronal membrane and efficiently labels fine terminal branches (Han et al., 2011). Similar to rodent studies using clinically relevant paclitaxel treatment paradigms, in the paclitaxel treatment paradigm described here, larval peripheral sensory axons did not show signs of degeneration (Fig. 2A1,B1). C4da axon terminals remained intact and synapsed in the VNC with a typical ladder-like pattern (Fig. 2A2,B2).

At 120 h AEL after 48 h exposure to paclitaxel, the area of the dendritic field defined by the dorsal projections of a single C4da neuron (Fig. 2A3,B3,C) was reduced proportionate to the restricted larval body size (Fig. 1F). Although the area of the receptive field was reduced in paclitaxel-treated larvae, dendritic coverage was properly scaled to match larval growth (Fig. S1C-E). Furthermore, dendritic tiling was still complete and there were no gaps between dendritic arbors of adjacent C4da neurons (Fig. S2). Interestingly, we observed a significant increase in terminal branches after paclitaxel treatment (Fig. 2D). When we adjusted for the size of the dendritic field, we found a marked 60% increase in terminal branch density (Fig. 2E) that was distributed throughout the receptive field as illustrated by Sholl analysis (Fig. 2F). To rule out the possibility of artifact from the CD4:tdGFP membrane reporter, we extended our analysis of paclitaxel-induced sensory phenotypes to *ppk-EGFP*, a cytosolic reporter widely used in studies of C4da nociceptors (Grueber et al., 2003). We confirmed that paclitaxel increased thermal sensitivity in larvae expressing *ppk-EGFP* (Fig. S3A-C). While EGFP incompletely labeled the arbor of C4da neurons, concomitant horseradish peroxidase (HRP) staining of neuronal membrane revealed supernumerary peripheral sensory dendrites in paclitaxel-treated larvae (Fig. S3D-E1).

We performed morphological analysis over the time course of treatment to uncover the root of increased dendrite branch density induced by paclitaxel. The area of the receptive field coordinated with larval growth and was proportionately suppressed by paclitaxel treatment (Figs 1F and 2A). At 96 h AEL we did not detect any differences in terminal dendrite branch numbers between treatment groups (Fig. 2B, Fig. S1A,B). There was a slight increase in the number of terminal branches with larval development from 96 to 120 h AEL in the vehicle-treated control group, and this increase was exacerbated by 48 h of exposure to paclitaxel (Fig. 2B). Dendrite branch density decreased over this developmental time period in vehicle-treated control larvae as a product of synchronized growth with body area (Fig. 2C). Paclitaxel-treated larvae exhibited time-dependent aberrant peripheral nociceptor branch density by 120 h AEL (Fig. 2C) as a product of slightly increased dendrite branching and constrained body growth.

**Fig. 3.**
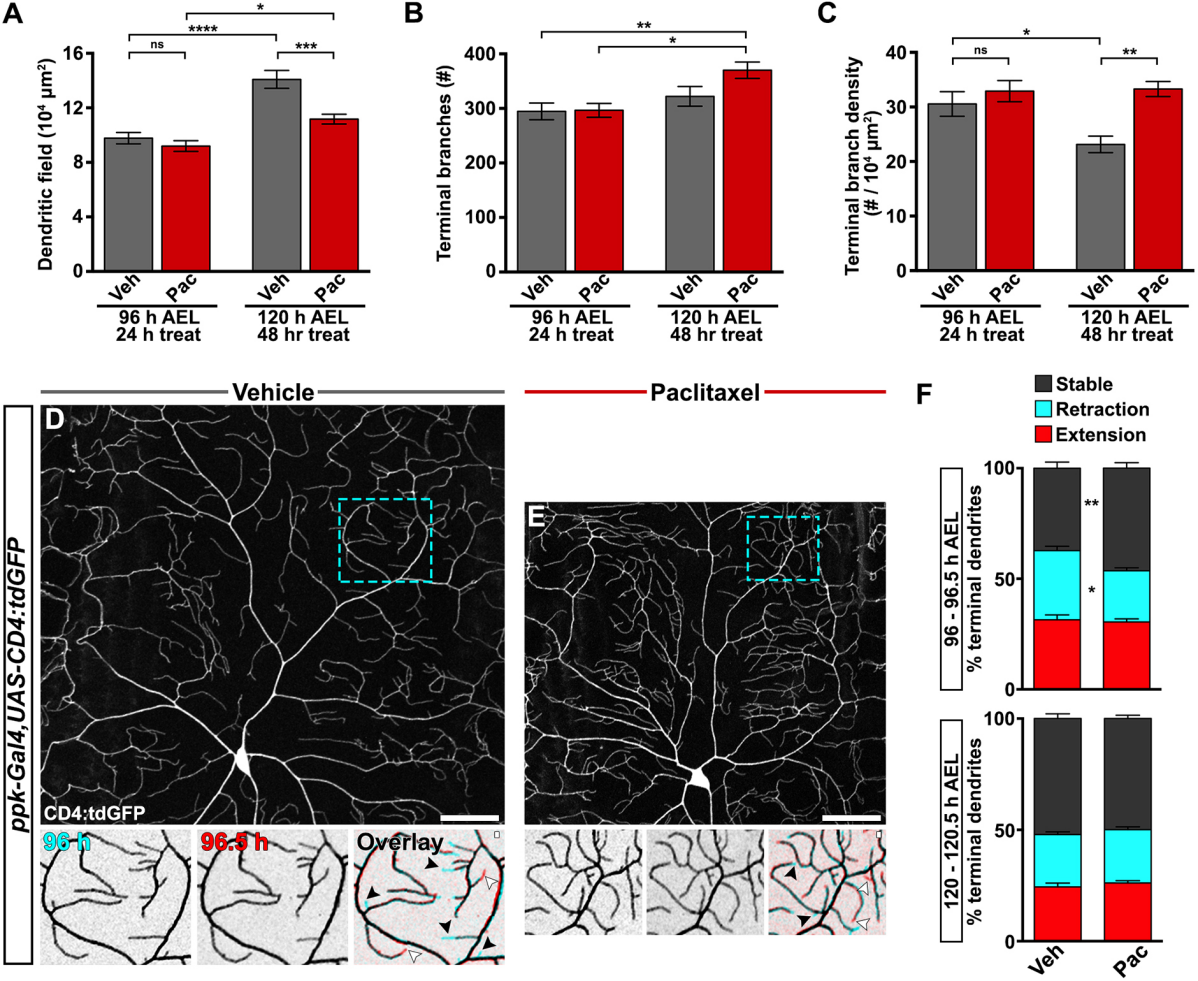
**Paclitaxel suppresses dendrite dynamics.** (A-C) Developmental and treatment time course of C4da dendrite field and terminal branching during treatment with either vehicle or paclitaxel quantified from CD4:tdGFP-labeled dendrites from confocal projections of fixed, filet-dissected larvae: (A) area of dendritic field measured in ImageJ, (B) number of terminal branches counted manually and (C) terminal branch density. Mean±s.e.m.; *n*≥7 neurons from >5 larvae for each time point and treatment, respectively; one-way ANOVA with Tukey's multiple comparisons test. (D,E) Dendrite terminal branch dynamics revealed by time-lapse imaging of C4da ddaC sensory dendrites labeled with CD4:tdGFP. Representative confocal projections of vehicle-treated (D) and paclitaxel-treated (E) larvae at 96 h AEL (top). Dashed box is magnified and shown below at 96 h and after a 30 min interval at 96.5 h. The overlay is pseudocolored so that the 96 h image is cyan and 96.5 h is red. In this way, stable dendrites are gray, retraction events are represented by cyan terminal dendrites and extension events are represented by red terminal dendrites. Black arrowheads indicate retraction events; white arrowheads indicate branch growth. Scale bars: 50 µm. (F) Relative percentages of stable terminal dendrites and terminal branches undergoing dynamic retraction or extension events over a 30 min interval at the 96-96.5 h time point (top) or 120-120.5 h time point (bottom) for vehicle- and paclitaxel-treated larvae. *n*=13 dendritic regions from >6 larvae at each time point and treatment. *P<0.05, **P<0.01, ***P<0.001, ****P<0.0001.

We next performed time-lapse imaging to uncover the cellular mechanisms involved in dendrite branch phenotypes induced by paclitaxel. We monitored dendrite dynamics at 30 m intervals and marked each terminal dendrite as either extending, retracting or stable. At 96 h AEL, over 60% of C4da nociceptor terminal branches had undergone dynamic rearrangements, with balanced extension and retraction events (Fig. 3D,F). At this same time period, paclitaxel treatment significantly reduced dendrite retraction and enhanced dendrite stability (Fig. 3E,F). Dendrites were relatively more stable at 120 h AEL regardless of treatment (Fig. 3F). Abnormal terminal branch stability is an early morphological phenotype induced by paclitaxel treatment, and is consistent with increased branching observed at the later stage in our treatment paradigm.

### Paclitaxel disrupts microtubule-associated Futsch organization in main branches of sensory dendrites

**Fig. 4.**
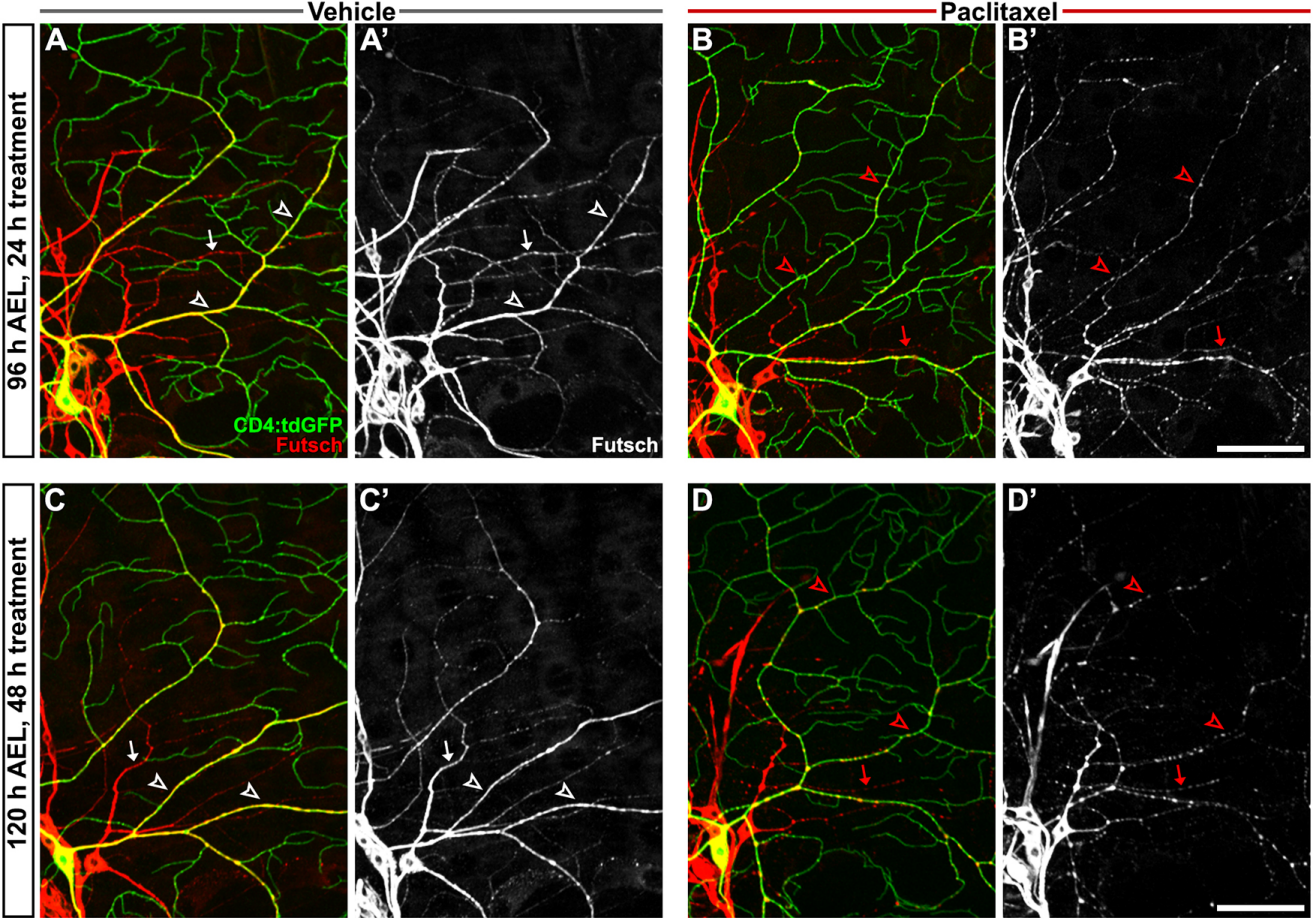
**Paclitaxel treatment disrupts microtubule-associated Futsch in sensory dendrites.** (A-D) Confocal projections showing immunostaining of the neuron-specific microtubule-associated protein Futsch within sensory dendrites from vehicle-treated (A,C) and paclitaxel-treated (B,D) larvae after 24 h (A,B) and 48 h (C,D) treatment. C4da neurons labeled by CD4:tdGFP exhibit a continuous Futsch staining pattern throughout the major proximal branches of vehicle-treated larvae (white arrowheads), whereas dendritic Futsch is disrupted upon paclitaxel treatment (red arrowheads) and appears progressively fragmented with treatment duration. Immunostaining also shows a similar disruption of Futsch continuity in other classes of peripheral neurons (arrows) by paclitaxel. Scale bars: 50 µm.

To investigate the *in vivo* effects of paclitaxel treatment on the microtubule cytoskeleton of peripheral sensory neurons, we genetically expressed a fluorescently tagged α-tubulin reporter (*UAS-GFP:α-tubulin*) together with a membrane-targeted fluorescent reporter (*UAS-CD4:tdTom*) within C4da neurons*.* GFP:α-tubulin appeared to distribute normally in larval sensory dendrites following paclitaxel treatment (Fig. S4). Since this reporter does not distinguish between tubulin monomers and those polymerized into microtubules, we next assessed endogenous microtubule organization by antibody staining for Futsch, a neuron-specific microtubule-associated protein and MAP1B homolog (Hummel et al., 2000). Futsch binds to α-tubulin and is an excellent marker of microtubules in major proximal dendrite branches of larval sensory neurons, consistent with the continuous staining pattern we observed in C4da dendrites of vehicle-treated larvae throughout the course of treatment (Fig. 4A,C). However, after 24 h of paclitaxel treatment, immunohistochemical analysis revealed fragmented and punctate Futsch within major dendritic branches of peripheral sensory neurons (Fig. 4B). Futsch fragmentation appeared to worsen with the length of treatment, and by 120 h there were more gaps and larger punctate areas of Futsch within dendrites (Fig. 4D). This phenotype was reproduced in paclitaxel-treated larvae expressing *ppk-EGFP* (Fig. S3D2). Our *in vivo* analysis suggests that paclitaxel disrupts the organization of the peripheral dendritic cytoskeleton but, as the terminal branches marked by either CD4:tdGFP reporter or HRP immunostaining were not labeled by either GFP:α-tub or Futsch, the resolution of our analysis does not extend to microtubule properties in the terminal branches.

### Nmnat is required for maintenance of sensory neuron integrity and function

**Fig. 5.**
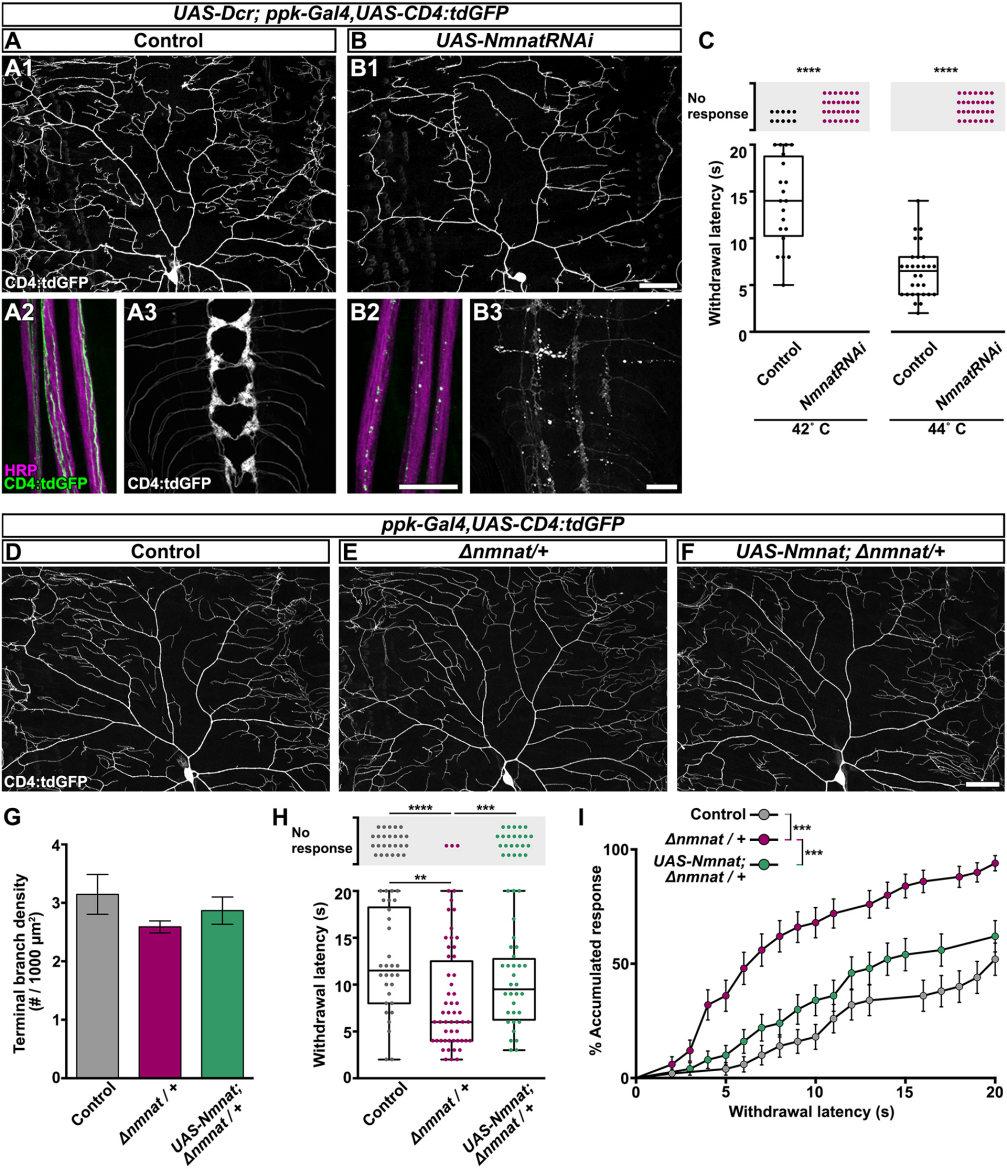
**Nmnat is required for maintenance of nociceptive integrity.** (A1-B3) Confocal projections of neuronal compartments labeled with CD4:tdGFP from control genotype (A) and larvae with RNAi-mediated C4da neuronal knockdown of Nmnat (*UAS-NmnatRNAi*; B) at 120 h AEL. Nmnat knockdown decreases dendritic branch complexity (B1), leads to axon degeneration (B2) and results in overt loss of synaptic terminals in the ventral nerve cord (B3). HRP labels all axonal membranes in peripheral nerve bundles (A2,B2). Dendrite scale bar: 50 µm; axon and VNC scale bars: 20 µm. (C) Withdrawal response of control genotype larvae and larvae with RNAi-mediated C4da Nmnat knockdown at 42 or 44°C stimulation. Each data point represents one larva. *n*=30 larvae tested at each temperature, Mann–Whitney test for response rate. (D-F) Representative confocal projections of C4da dorsal dendrites labeled with CD4:tdGFP from larvae of control genotype (D), heterozygous for *Nmnat* (E; *Δnmnat/+*), and heterozygous for Nmnat with C4da overexpression of Nmnat (F; *UAS-Nmnat; Δnmnat/+*) at 120 h AEL. Scale bar: 50 µm. (G) Quantification of terminal branch density from larvae of control genotype, *Nmnat* heterozygotes, and *Nmnat* heterozygotes with C4da overexpression of Nmnat. Mean±s.e.m.; *n*≥6 neurons from >4 larvae, one-way ANOVA. (H) Withdrawal response of larvae of control genotype, *Nmnat* heterozygotes, and *Nmnat* heterozygotes with C4da overexpression of Nmnat at 42°C stimulation. *n*=55 larvae tested from each group, Kruskal–Wallis with Dunn's multiple comparisons test for response rate and mean withdrawal latency. **P<0.01, ***P<0.001, ****P<0.0001. (I) Cumulative distribution of latency curve of larval withdrawal responses in H. This graph plots the accumulated percent response as a function of withdrawal latency, providing a curve that fits all of the larvae tested, both those that respond aversely and those that do not perceive noxious stimulation within the 20 s cutoff. Each pair of groups is initially compared by log-rank (Mantel–Cox) test and adjusted for multiple comparisons by Bonferroni-corrected threshold (Bonferroni-corrected threshold=family-wise significance level/*K*, where *K* is the number of comparisons). When the *P*-value of Mantel–Cox test comparing two curves is <the Bonferroni-corrected threshold of 0.05/3=0.0167 or 0.001/3=0.00033, the comparison is considered statistically significant. ***P<0.00033.

With the goal of identifying modulators of sensory symptoms relevant to paclitaxel-induced CIPN, we first asked whether the neuronal maintenance factor Nmnat influences sensory function by loss-of-function analysis. In mammals, there are three NMNAT isoforms. The axonal isoform, NMNAT2, is required at levels above 25% of wild-type levels for normal neuronal development and maintenance (Gilley et al., 2013). We have previously shown that the sole *Drosophila* Nmnat is not required for neuronal development, but is essential for neuronal maintenance, including C4da neuronal processes (Wen et al., 2011; Zhai et al., 2006). When we knocked down Nmnat in C4da neurons (*UAS-Dcr; ppk-Gal4, UAS-CD4:tdGFP/UAS-NmnatRNAi*), we observed a striking reduction of dendrite complexity and coverage relative to control (Fig. 5A1-B1). We also observed pronounced degeneration of sensory axons and synaptic terminals (Fig. 5A2-B3). Not surprisingly, the extensive loss of neuronal processes observed upon RNAi-mediated Nmnat depletion corresponded to a complete loss of thermal perception at 42°C stimulation (Fig. 5C). Complete insensitivity was also observed at 44°C stimulation, where all control larvae respond aversely (Fig. 5C). Based on our immunohistochemical analysis of the nuclear region of peripheral sensory neurons, C4da RNAi-mediated knockdown depletes Nmnat protein levels by ∼80% of control C4da neurons (ddaC neurons, Fig. S5A-C); nearby C1da neurons (ddaE neurons), express normal Nmnat protein amounts, demonstrating cell specificity of the approach. As with mammalian NMNAT2 (Gilley and Coleman, 2010), *Drosophila* Nmnat must then exceed this threshold level to fulfill neuronal maintenance requirements.

To avoid the degenerative effects of RNAi-mediated Nmnat knockdown, we next investigated sensory neuron maintenance in larvae carrying one *Nmnat* deletion allele (*Δnmnat*), which results in a 50% reduction of Nmnat protein within all peripheral neurons (Fig. S5D-F). We have previously shown that *Δnmnat/+* heterozygous larvae exhibit a progressive reduction in C4da dendrite branch density (Wen et al., 2011), but this study utilized the same cytosolic reporter (*ppk-EGFP*) that obscured paclitaxel-induced dendrite defects here (Fig. S3). By visualizing the robust CD4:tdGFP membrane-targeted reporter, we determined that the morphology and branch density of C4da neurons in *Δnmnat/+* heterozygous larvae were indistinguishable from controls (Fig. 5D-G). Yet, when we examined nociceptive function, we found that significantly more *Δnmnat/+* heterozygous larvae exhibited aversive withdrawal from 42°C stimulation, and that these larvae exhibited a 33% decrease in latency compared to controls (Fig. 5H). Return-of-function C4da expression of Nmnat significantly restored nociceptive response rate (Fig. 5H). We also present these behavioral data in a cumulative distribution of latency curve (Im et al., 2015) depicting percent accumulated response as a function of withdrawal latency (Fig. 5I). This allows us to compare the sensitivity of each group holistically by providing a curve that considers all of the larvae tested, both those that respond aversely and those that do not perceive noxious stimulation within the 20 s cutoff. Collectively, these results suggest that peripheral nociceptors are sensitive to the levels of Nmnat and unveil a complex relationship between dendrite morphology and nociceptive function.

**Fig. 6.**
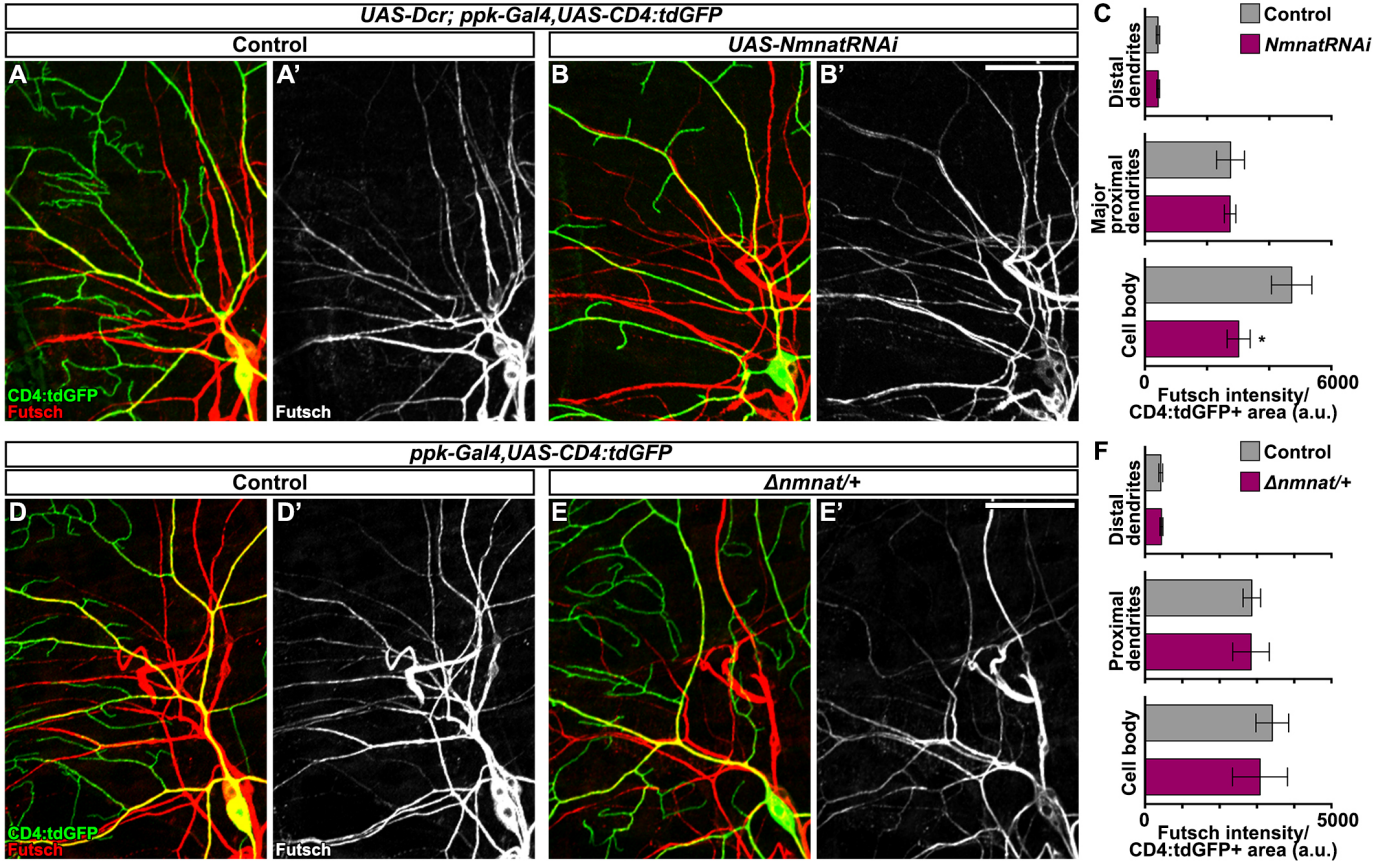
**Dendritic Futsch organization does not require Nmnat.** (A-B′) Confocal projections showing immunostaining of microtubule-associated protein Futsch within sensory dendrites from larvae with C4da neuronal RNAi-mediated knockdown of Nmnat (B; *ppk-Gal4>UAS-Dcr,UAS-NmnatRNAi*) and controls (A; *ppk-Gal4>UAS-Dcr*). C4da neurons exhibit continuous Futsch staining throughout the major branches, even in those with substantial dendrite loss (B). Scale bar: 50 µm. (C) Futsch intensity measured within C4da neuronal compartments, i.e. cell body, major proximal dendrite branches and distal dendrite branches, in larvae with C4da RNAi-mediated knockdown of Nmnat and controls. Mean±s.e.m.; *n*=5 neurons from >4 larvae from each genotype, Student's *t*-test. (D-E′) Confocal projections of Futsch immunostaining and C4da expression of CD4:tdGFP in *Nmnat* heterozygous larvae (E; *Δnmnat/+*) and controls. (F) Futsch intensity measured within C4da neuronal compartments in larvae heterozygous for *Nmnat* and controls. Mean±s.e.m.; *n*=5 neurons from >4 larvae from each genotype, Student's *t*-test. *P<0.05.

*Δnmnat/+* larvae exhibit sensory dysfunction reminiscent of paclitaxel-induced hypersensitivity, without similar nociceptor dendrite density phenotypes. We also probed for Futsch to reveal possible shared mechanisms underlying peripheral sensory neuron-mediated hypersensitivity. However, unlike Futsch fragmentation in major dendritic branches upon paclitaxel treatment, we found that organization and continuity of Futsch within sensory dendrites was unaffected by Nmnat loss of function either by RNAi or copy number reduction approaches (Fig. 6A,B,D,E). We did observe reduced levels of Futsch protein in the sensory neuron cell bodies upon RNAi-mediated Nmnat knockdown (Fig. 6C), but levels within the major proximal branches were unchanged (Fig. 6C,F).

### Nmnat overexpression mitigates paclitaxel-induced nociceptive hypersensitivity

**Fig. 7.**
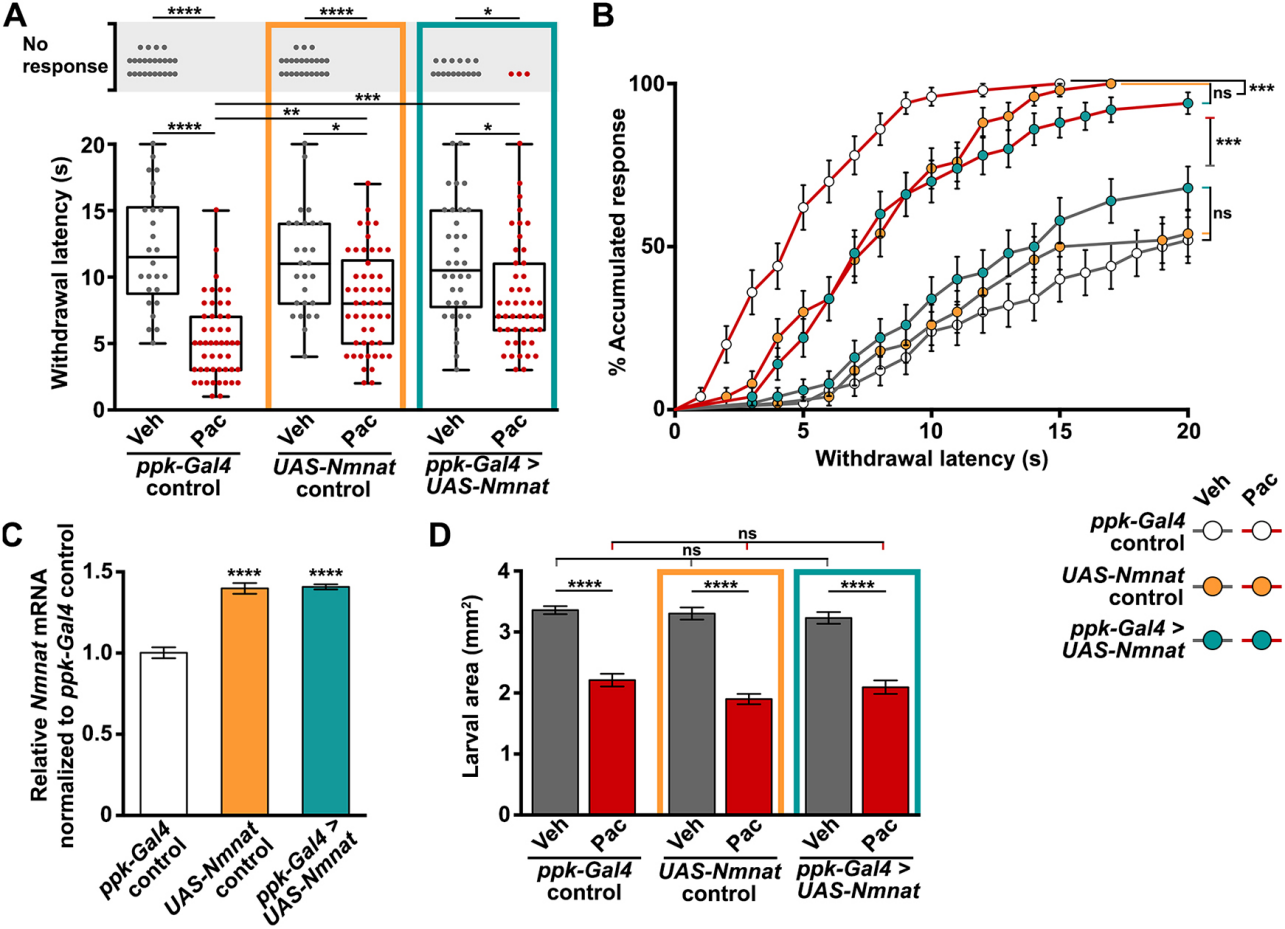
**Nmnat mitigates paclitaxel-induced sensory dysfunction.** (A) Withdrawal response of *ppk-Gal4* control larvae, *UAS-Nmnat* control and larvae overexpressing Nmnat in C4da sensory neurons (*ppk-Gal4>UAS-Nmnat*) after 48 h exposure to either vehicle or paclitaxel. *n*=50 larvae tested for each genotype and treatment, response rate: Kruskal–Wallis with Dunn's multiple comparisons test, mean withdrawal latency: one-way ANOVA with Tukey's multiple comparisons test. (B) Cumulative distribution of latency curve representative of all larvae tested in A plotted as % accumulated response as a function of withdrawal latency. Mantel–Cox test and adjusted for multiple comparisons by Bonferroni-corrected threshold, here 0.001/9=0.00011. ***P<0.00011. (C) *Nmnat* mRNA levels measured from whole larvae of all genotypes at 120 h AEL (*ppk-Gal4* control, *UAS-Nmnat* control, and Nmnat overexpression in C4da sensory neurons *ppk-Gal4>UAS-Nmnat*) by quantitative real-time PCR expressed relative to the housekeeping gene *Rp49* and normalized to *ppk-Gal4* control. Mean±s.e.m.; *n*=4 independent tests of 5-10 larvae from each group. (D) Effect of paclitaxel treatment on growth of *ppk-Gal4* control larvae, *UAS-Nmnat* control and larvae overexpressing Nmnat in C4da sensory neurons (*ppk-Gal4>UAS-Nmnat*). Larval body area was calculated as the length multiplied by the width of each larva measured in ImageJ from images acquired from a dissection microscope. Mean±s.e.m.; one-way ANOVA with Tukey's multiple comparisons test. *P<0.05, **P<0.01, ***P<0.001, ****P<0.0001.

Nmnat overexpression has been shown to be neuroprotective against paclitaxel-induced degeneration in both mammals and *Drosophila* (Bhattacharya et al., 2012; Wang et al., 2002). We asked whether C4da neuronal overexpression of Nmnat could ameliorate hypersensitivity associated with dendrite stability and aberrant density observed in larvae subjected to our paclitaxel treatment paradigm. Indeed, paclitaxel-induced thermal hypersensitivity was partially relieved by overexpressing Nmnat within C4da primary nociceptors, yet was equally alleviated by the presence of the *UAS-Nmnat* transgene without the C4da neuron-specific *ppk-Gal4* driver (mean withdrawal latency in s ±s.e.m. after 48 h paclitaxel treatment: *ppk-Gal4* control=5.2±0.9; *UAS-Nmnat* control=8.2±0.5, *ppk-Gal4>UAS-Nmnat*=8.4±0.6; Fig. 7A). The accumulated response curve clearly illustrates that although paclitaxel engendered hypersensitivity in all groups, the *UAS-Nmnat* transgene still significantly mitigated the severity of sensory dysfunction induced by paclitaxel treatment (Fig. 7B). We performed quantitative real-time PCR and found that the *UAS-Nmnat* transgene results in a 20% global upregulation of *Nmnat* mRNA relative to *ppk-Gal4* controls (Fig. 7C). Nmnat overexpressed specifically in C4da neurons was undetectable above basal leaky expression by this approach. It is important to note that the effect of Nmnat on sensory function was not a consequence of larval size, because paclitaxel treatment inhibited larval growth similar to *ppk-Gal4* controls (Fig. 7D). Together, these data suggest that the broad overexpression of Nmnat can mitigate paclitaxel-induced thermal hypersensitivity.

**Fig. 8.**
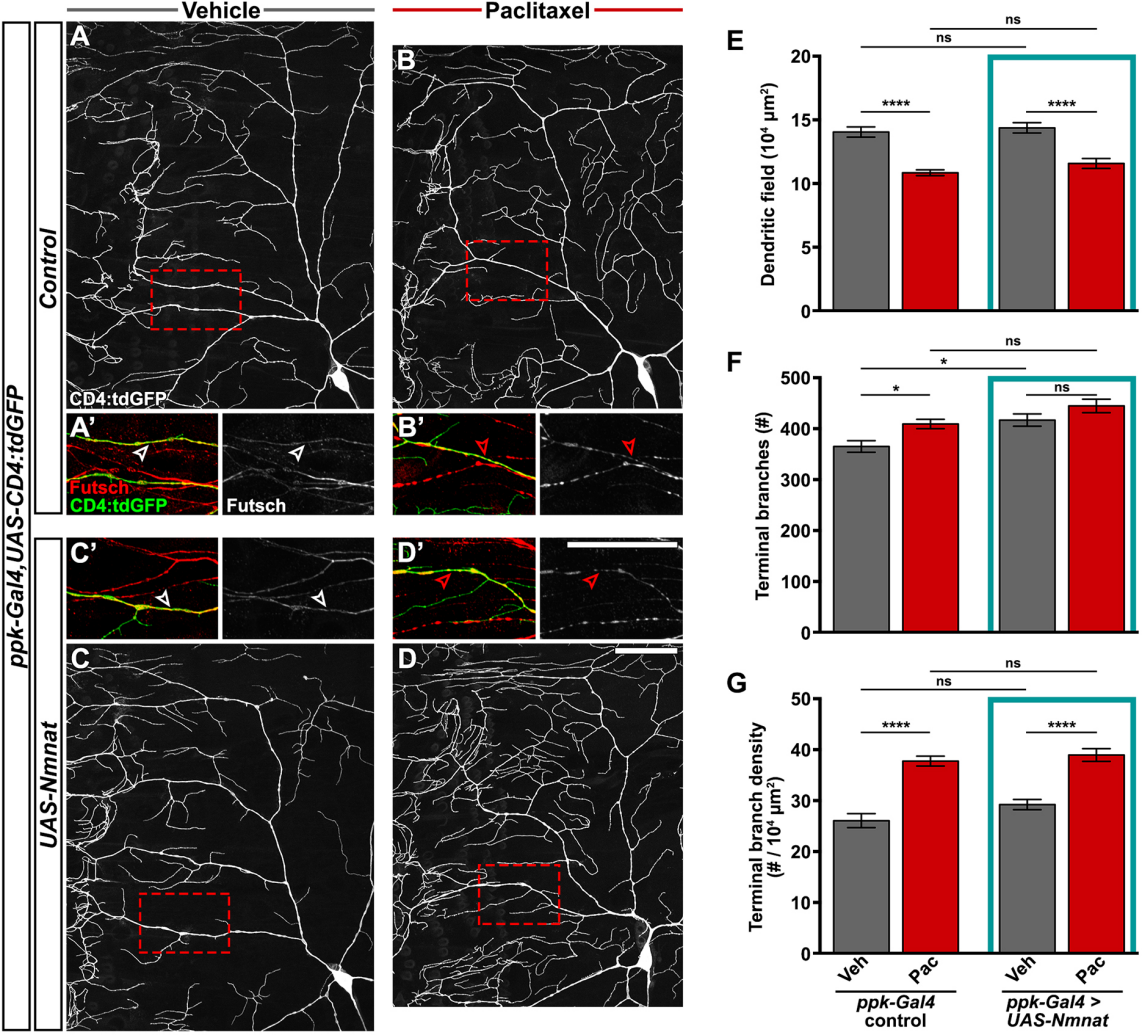
**Nmnat does not prevent paclitaxel-induced dendrite density or Futsch disruption.** (A-D) Confocal projections showing ddaC sensory dendrites labeled with CD4:tdGFP from control genotype treated with vehicle (A) or paclitaxel (B), and larvae overexpressing Nmnat in C4da sensory neurons (*UAS-Nmnat*) treated with vehicle (C) or paclitaxel (D). Scale bar: 50 µm. (A′-D′) High magnification of dendritic regions boxed in A-D, showing immunostaining of microtubule-associated Futsch in C4da dendrites (arrowheads) and other classes of peripheral neurons. Scale bar: 50 µm. (E-G) Quantification of dendritic field (E), number of terminal branches (F) and terminal branch density (G) of the dorsal projections of ddaC neurons from *ppk-Gal4* control larvae and larvae overexpressing Nmnat in C4da sensory neurons after 48 h treatment with vehicle or paclitaxel. Means±s.e.m.; *n*=14 neurons from >7 larvae form each genotype and treatment; one-way ANOVA with Tukey's multiple comparisons test. *P<0.05, ****P<0.0001; ns, nonsignificant.

We next addressed the effect of Nmnat expression on dendrite alterations after paclitaxel treatment. Interestingly, paclitaxel treatment increased dendrite branch density independent of Nmnat overexpression (Fig. 8A-D,G). Paclitaxel treatment also disrupted Futsch staining in C4da dendritic branches independent of Nmnat overexpression (Fig. 8A′-D′). Thus, Nmnat is likely to be acting in a pathway parallel to or downstream of Futsch organization and dendrite branch density to ameliorate sensory dysfunction in our model of paclitaxel-induced peripheral neuropathy.

## DISCUSSION

Our study proposes new insights into CIPN and sensory dysfunction induced by the microtubule-stabilizing agent paclitaxel using an improved *Drosophila* larval model. Analogous to symptoms of patients suffering from CIPN, larvae fed paclitaxel develop dose- and duration-dependent hypersensitivity to noxious thermal stimulation (i.e. hyperalgesia) and display a lower threshold for thermal tolerance (i.e. allodynia). In our model, paclitaxel exposure enhances stability of the normally dynamic nociceptive sensory endings leading to increased terminal density, which, to our knowledge, would represent a novel cellular mechanism in CIPN and one that is congruent with a thermal nociceptive gain of function. We found that the neuronal maintenance factor Nmnat is protective against paclitaxel-induced sensory dysfunction and that endogenous Nmnat is a critical determinant of peripheral nociceptive sensitivity. Our work establishes a robust and tractable model for studying peripheral mechanisms of CIPN and highlights Nmnat's potential role as a modulator of sensory dysfunction in CIPN.

### An *in vivo* model: dose-dependent pathology and dysfunction

CIPN poses negative impacts on cancer outcomes and quality of life for survivors, and thus demands research into effective neuroprotective and symptomatic treatment strategies. Although *in vitro* and cell culture studies have afforded invaluable chemical, molecular and cellular evidence regarding mechanism of action of chemotherapy drugs, *in vivo* models are absolutely critical to fully understand how these mechanisms engender neurotoxicity and induce sensory dysfunction. Preclinical studies using traditional rodent models have been complicated by inconsistencies in paclitaxel dosing and administration (Hopkins et al., 2016). Nevertheless, the emergent trend indicates that paclitaxel-induced neuropathology and sensory dysfunction are dose dependent. High doses of paclitaxel lead to degeneration of peripheral sensory axons associated with diminished physiological and behavioral measures of sensory function (Authier et al., 2000; Cavaletti et al., 1997; Wang et al., 2002); low-dose treatment schedules representative of clinical regimens correlate with positive sensory symptoms, but are largely devoid of morphological signs of axon degeneration (Flatters and Bennett, 2006; Polomano et al., 2001). Consistent with the dose-dependent nature of paclitaxel-induced neurotoxicity, a previous study reported neuronal degeneration in *Drosophila* larvae after prolonged high-dose paclitaxel feeding (Bhattacharya et al., 2012).

The optimal preclinical model should exhibit an appropriate level of neuropathology yet still engender functional sensory disturbances relevant to patients suffering with CIPN. We have designed our *Drosophila* larval model accordingly. First, we outlined a moderate paclitaxel feeding schedule, upon which *Drosophila* larvae develop hypersensitivity to noxious thermal stimulation without signs of peripheral nerve degeneration. Second, we selected a stimulation temperature that affords the most dynamic range and measurement resolution for assessing treatment-induced changes in thermal nociceptive sensitivity, whether manifested as sensory deficits or hypersensitivity. Finally, we employed a cumulative distribution of latency curve to quantitate nociceptive behavior, accounting for both the withdrawal latency and the response rate, which is essential for the comprehensive analysis of the nociceptive response across a population of larvae. These improvements have optimized the model to allow for mechanistic study of paclitaxel-induced sensory dysfunction as well as for screening potential therapeutic modifiers.

### A cellular mechanism: terminal sensory endings

Paclitaxel-induced sensory disturbances present with a ‘glove and stocking’ distribution, long postulated as a consequence of length-dependent axon degeneration owing to the extensive distance between sensory neuron cell bodies in the dorsal root ganglia (DRGs) and their terminal endings in the hands and feet (Fukuda et al., 2017). However, in *Drosophila* larvae, sensory dendrites elaborate directly from the neuronal cell bodies in the periphery. The sensory dendrite pathology observed in our larval model implicates an inherent susceptibility of the sensory terminal endings to paclitaxel exposure. Sensory terminal branches innervating the epidermis are highly dynamic and remodel their fine endings as skin cells turn over (Cheng et al., 2010). Similarly, *Drosophila* terminal sensory dendrites are dynamic, exhibiting retraction and extension events (Parrish et al., 2009). Here, we demonstrate that paclitaxel promotes terminal dendrite stability and inhibits branch retraction. In a CIPN rat model, paclitaxel led to decreased density of intraepidermal nerve fibers (IENFs; the terminal arbor of sensory axons) innervating the skin (Bennett et al., 2011). Using microfluidic chambers, it was shown that local application of paclitaxel in the distal axon, but not the mid axon or cell body compartment, of cultured DRG neurons prevented neurite outgrowth (Gornstein and Schwarz, 2017). Taken together, paclitaxel may inhibit terminal sensory ending plasticity within the specific context of the microenvironment, i.e. terminal dendrite refinement during larval development, IENF terminal arbor remodeling during epidermal renewal, or growth cone dynamics during the regenerative state of cultured neurons. Our work highlights peripheral sensory endings that innervate the epidermis as a particularly vulnerable compartment to paclitaxel, perhaps underlying the predominantly distal sensory symptoms observed in patients suffering from CIPN.

The microtubule cytoskeleton underlying *Drosophila* C4da sensory dendrites is critically important for dendrite outgrowth and stabilization, and ultimately for proper nociceptive function. Multiple microtubule regulatory mechanisms have been shown to contribute to shaping the dendritic arbor, including microtubule severing enzymes (Jinushi-Nakao et al., 2007; Stewart et al., 2012), tubulin post-translational modifications (Jenkins et al., 2017) and microtubule nucleation from Golgi outposts (Ori-McKenney et al., 2012). Similarly, we have shown that paclitaxel can regulate dendrite branching, yet the direct mechanistic link to sensory dysfunction is still unclear. By immunofluorescence labeling, we revealed that paclitaxel disrupts the continuity and integrity of the microtubule-associated protein Futsch/MAP1B within peripheral sensory dendrites. Interestingly, larvae mutant for *futsch* exhibit increased dendritic branching of peripheral sensory neurons (Jenkins et al., 2017; Yalgin et al., 2015), and MAP1B-deficient cultured DRG neurons show a pronounced increase in terminal and collateral branching (Bouquet et al., 2004). These studies suggest that Futsch/MAP1B, microtubule stability and dendritic processes are all intricately linked. MAP1B has also been implicated in synaptic plasticity and maturation (Tortosa et al., 2011), and has been shown to play a distinct role in receptor internalization during activity-dependent synaptic depression, likely through regulation of the actin cytoskeleton (Benoist et al., 2013). Futsch/MAP1B may then be a key link between microtubule-mediated dendrite stability and dendrite-mediated sensory processing.

### A neuroprotective modulator: Nmnat

Nmnat overexpression has been utilized extensively as a tool to define degeneration in models of neuronal injury and neurodegenerative disorders, where Nmnat-mediated protection is considered to be indicative of mechanistic overlap with the Wallerian degeneration axon destruction pathway. As Nmnat overexpression has been shown to robustly protect axons against a wide variety of insults (Conforti et al., 2014), it might be expected that Nmnat would alleviate signs and symptoms of paclitaxel neuropathy associated with axon degeneration. Indeed, the Wld^S^ (Wallerian degeneration slow) mouse, which harbors a mutation causing overexpression of an Nmnat1 fusion protein, was shown to be protected from paclitaxel-induced behavioral and electrophysiological deficits associated with sensory axon degeneration (Wang et al., 2002). In *Drosophila* larvae, a paclitaxel-induced axon injury paradigm was devised to identify conserved genes in the axon degeneration pathway (Bhattacharya et al., 2012); Nmnat overexpression delayed C4da sensory axon and dendrite degeneration. Recently, it was shown that mice lacking the pro-degenerative molecule Sarm1, an activator of the Wallerian degeneration pathway, are resistant to paclitaxel-induced IENF loss and corresponding sensory deficits (Turkiew et al., 2017). Depletion of Nmnat is sufficient to induce Wallerian degeneration (Gilley and Coleman, 2010), and consistently we show that RNAi-mediated depletion of *Drosophila* Nmnat causes neurodegeneration associated with a complete absence of thermal nociception. These studies corroborate shared pathways underlying Wallerian degeneration and paclitaxel-induced neuronal degeneration, and also establish a clear link between peripheral sensory neuron degeneration and sensory deficits.

Less clear is the role of Nmnat in the context of paclitaxel treatment associated with hypersensitivity. In mammals, three Nmnat isoforms (Nmnat1-3) are preferentially expressed in different tissues and subcellular compartments. Endogenous Nmnat2 is enriched in the nervous system and is required for neuronal survival (Gilley and Coleman, 2010). Mice heterozygous for Nmnat2 develop paclitaxel-induced hypersensitivity at the same rate and extent as controls (Slivicki et al., 2016). It should be noted that *Nmnat2* heterozygous mice did not show signs of hypersensitivity at baseline, possibly owing to genetic redundancy of Nmnat. We did not examine the double insult of Nmnat deficiency together with paclitaxel treatment, but rather focused on the neuroprotective aspect of Nmnat overexpression. In our study, the sole *Drosophila* Nmnat partially protected larvae from developing thermal hypersensitivity after paclitaxel treatment. Impressively, Nmnat preserved neuronal function even in the presence of cellular and molecular defects, i.e. increased sensory dendrite branch density and disruption of microtubule-associated Futsch. Similarly, our investigations into endogenous Nmnat requirements showed that Nmnat heterozygous larvae also developed nociceptive hypersensitivity without any morphological indications. This uncoupling of ‘form and function’ was surprising but not unprecedented; in a screen for genes required for thermal nociception in *Drosophila* larvae, C4da branching was found to be poorly correlated with sensitivity (Honjo et al., 2016). Further studies are necessary to establish the molecular basis of Nmnat-mediated protection and maintenance, but a compelling hypothesis is that Nmnat promotes microtubule dynamics to counteract the microtubule-stabilizing properties of paclitaxel; microtubule dynamics are upregulated in dendrites after axon injury in an Nmnat-dependent manner (Chen et al., 2016). These investigations are warranted because Nmnat has the remarkable capacity to alleviate paclitaxel-induced sensory symptoms ranging from sensory loss associated with axon degeneration to hypersensitivity associated with increased dendrite density, thus is poised to effectively alleviate the spectrum of symptoms experienced by patients suffering from CIPN.

## MATERIALS AND METHODS

### *Drosophila* stocks and genetics

Fly stocks were maintained at 25°C in a 12 h light:12 h dark cycle. The y^1^w* strain was used for all control crosses. *nmnatΔ4790-1* and *UAS-Nmnat* (Zhai et al., 2006), *ppk-EGFP* (Grueber et al., 2003) and *ppk-Gal4* (Grueber et al., 2007) are as previously described. *UAS-CD4:tdGFP* (stock 35836), *UAS-CD4:tdTom* (stock 35837), *UAS-GFP:α-tubulin* (stock 7373) and *UAS-NmnatRNAi* (stock 29402) flies were obtained from the Bloomington Stock Center (Bloomington, IN).

### Thermal nociceptive withdrawal assay

Larvae were rinsed in water and gently placed on a glass slide where they were allowed to acclimate for 10 s. Thermal stimuli were administered using a custom-built thermal probe based on a previously reported design (Babcock et al., 2009). An operator blind to genotype and treatment gently stimulated larvae laterally between abdominal segments A3-A5 under a light microscope. Withdrawal response was defined as time to initiate aversive corkscrew-like rolling behavior and recorded as withdrawal latency up to a 20 s cutoff or otherwise categorized as no response. Environmental factors such as lighting, time of day and room temperature were held constant throughout the experiment. Survival analysis in GraphPad was used to plot and analyze the cumulative distribution of latency curves. Each subject larva was entered on a separate row in the table; larvae that did not respond within 20 s were ‘censored’ at that time and the withdrawal latency was recorded for responders.

### Paclitaxel treatment

Virgin parental flies (∼50 females and ∼20 males) were mated for 48-72 h and transferred to embryo collection cages consisting of perforated 100 ml plastic beakers covering 60 mm grape juice agar plates (for 50 plates of ∼4 ml: 150 ml ddH_2_0, 50 ml grape juice concentrate, 6 g agar, 11 g sucrose, 5 ml ethanol, 2.5 ml acetic acid) supplemented with yeast paste (700 mg baker's yeast in 1 ml ddH_2_0). Embryos were collected for 2-4 h and allowed to develop for 72 h in a 25°C incubator. Paclitaxel (5 mg; Sigma-Aldrich, St Louis, MO) was dissolved in DMSO to achieve stock concentration of 10 mM that was stored in single-use aliquots at −20°C. Grape juice agar plates for paclitaxel treatment were made fresh by adding stock paclitaxel (20 µM final concentration, or 10, 20 and 30 µM for dose-response experiment) or DMSO (0.2% final) to warm grape juice agar solution and mixing thoroughly. Grape juice agar solution with paclitaxel or vehicle was poured into 60 mm plates and allowed to cool before supplementing with yeast paste containing equivalent concentrations of paclitaxel or vehicle, respectively. At 72 h AEL, 100 third-instar larvae were rinsed in water, divided into groups of 50 and transferred to grape juice agar plates containing paclitaxel or vehicle only. Larvae were returned to the incubator for the remaining time course of treatment.

### Immunohistochemistry

Larvae were filleted along the ventral midline and pinned flat on a Sylgard dissection plate filled with phosphate-buffered saline (PBS). Larvae were fixed for 35 min at room temperature in 4% formaldehyde in PBS followed by 4×10 min washes in PBST (0.3% Triton X-100 in PBS) with gentle agitation. Larvae were transferred to 0.5 ml tubes and blocked in 5% normal goat serum in PBST for 1 h. Antibody solutions were prepared in blocking solution. Samples were incubated with primary antibody overnight at 4°C then washed 4×15 min in PBST at room temperature. Samples were incubated with secondary antibodies for 2 h at room temperature then washed 4×15 min in PBST. Primary antibodies were: mouse IgG1 anti-Futsch (22C10) used at 1:500 and mouse IgG2a anti-FasIII (7G10) used at 1:100 (Developmental Studies Hybridoma Bank, University of Iowa, IA), goat anti-HRP-Cy5 used at 1:500 (Jackson ImmunoResearch Laboratories, West Grove, PA), and guinea pig anti-Nmnat used at 1:500 (Zhai et al., 2006). Secondary antibodies used at 1:250 dilutions were: Alexa-Fluor-555 anti-mouse for Futsch visualization, Alexa-Fluor-555 anti-guinea pig for Nmnat visualization, and Alexa-Fluor-647 anti-mouse IgG1 and Alexa-Fluor-568 anti-mouse IgG2a for Futsch and FasIII co-staining (Invitrogen). Samples were mounted in Vectashield Antifade mounting medium (Vector Laboratories, Burlingame, CA).

### Neuron visualization and analysis

Confocal image stacks were obtained using an Olympus FluoView FV1000 microscope and converted to maximum intensity projections using ImageJ software (NIH, Bethesda, MD). The dorsal projection of one C4da ddaC neuron per larva from abdominal segment 3 or 4 was used for analysis. All analyses were performed on a rectangular region of interest (ROI) drawn to encompass the dorsal half of the dendritic arbor from the cell body to the midline. Dendritic area was measured in ImageJ and the number of terminal dendritic branches was assessed by manual counts by a blind observer. Sholl analysis (Ferreira et al., 2014) was performed in ImageJ on threshold-segmented dendritic arbors with the relative radii defined by the length of the enclosing radius drawn orthogonally anterior to the cell body divided by 20. Futsch intensity was measured in ImageJ within C4da neuronal compartments by applying a mask generated from the CD4:tdGFP channel. ROIs were selected near the soma for proximal dendrites or near the dorsal midline for distal dendrites by an observer blind to genotype. For Nmnat RNAi and controls, proximal dendrite analysis was restricted to the major branches by manually eliminating higher-order branches from the mask. Nmnat protein levels were measured from a single *z* plane through the nucleus of a C4da ddaC neuron and the C1da ddaE neuron from the same dorsal cluster. An ROI was drawn around the nucleus of each neuron using DAPI as a landmark, and fluorescence intensity was measured from the Nmnat channel and normalized to the ROI area for Nmnat density.

### Quantitative real-time PCR

Independent experiments of 5-10 larvae from each group were stored at −80°C before homogenizing in TRI Reagent (Sigma-Aldrich). Total RNA was isolated by phenol-chloroform extraction method and purified by Total RNA Purification Mini Kit (Favorgen Biotech, Taiwan, China). RNA concentration was measured by spectrophotometer (BMG LABTECH, NC, USA) at 260 nm and a total amount of 2 µg RNA was used for reverse transcription to cDNA using a High-Capacity cDNA Reverse Transcription Kit (Thermo Fisher Scientific Baltics, Vilnius, Lithuania). Quantification of mRNA levels was performed by SYBR-Green-based gene expression analysis using a CFX connect real-time PCR detection system (Applied Biosystems, CA, USA). The samples were amplified by 40 cycles of 15 s at 95°C and 1 min at 60°C. Housekeeping gene *Rp49* was used as a loading control. The primer sequences are as follows (5′-3′): *Nmnat* (forward): GGCCAGTCGGTCAAGTACC, *Nmnat* (reverse): CGGTGCATCCCGCGATTT, *Rp49* (forward): CTAAGCTGTCGCACAAATGGC, *Rp49* (reverse): AACCGATGTTGGGCATCAGA.

### Statistical analysis

Statistical tests were performed in GraphPad Prism as indicated in the figure legends.

## Supplementary Material

Supplementary information
