# Supplementary material for: Nmnat mitigates sensory dysfunction in a Drosophila model of paclitaxel-induced peripheral neuropathy
Source: Dis Model Mech. 2018 Jun 12;11(6):dmm032938. doi: 10.1242/dmm.032938 (PMC6031360; doi:10.1242/dmm.032938)
Supplement: Supplementary information [file dmm-11-032938-s1.pdf]

Supplementary

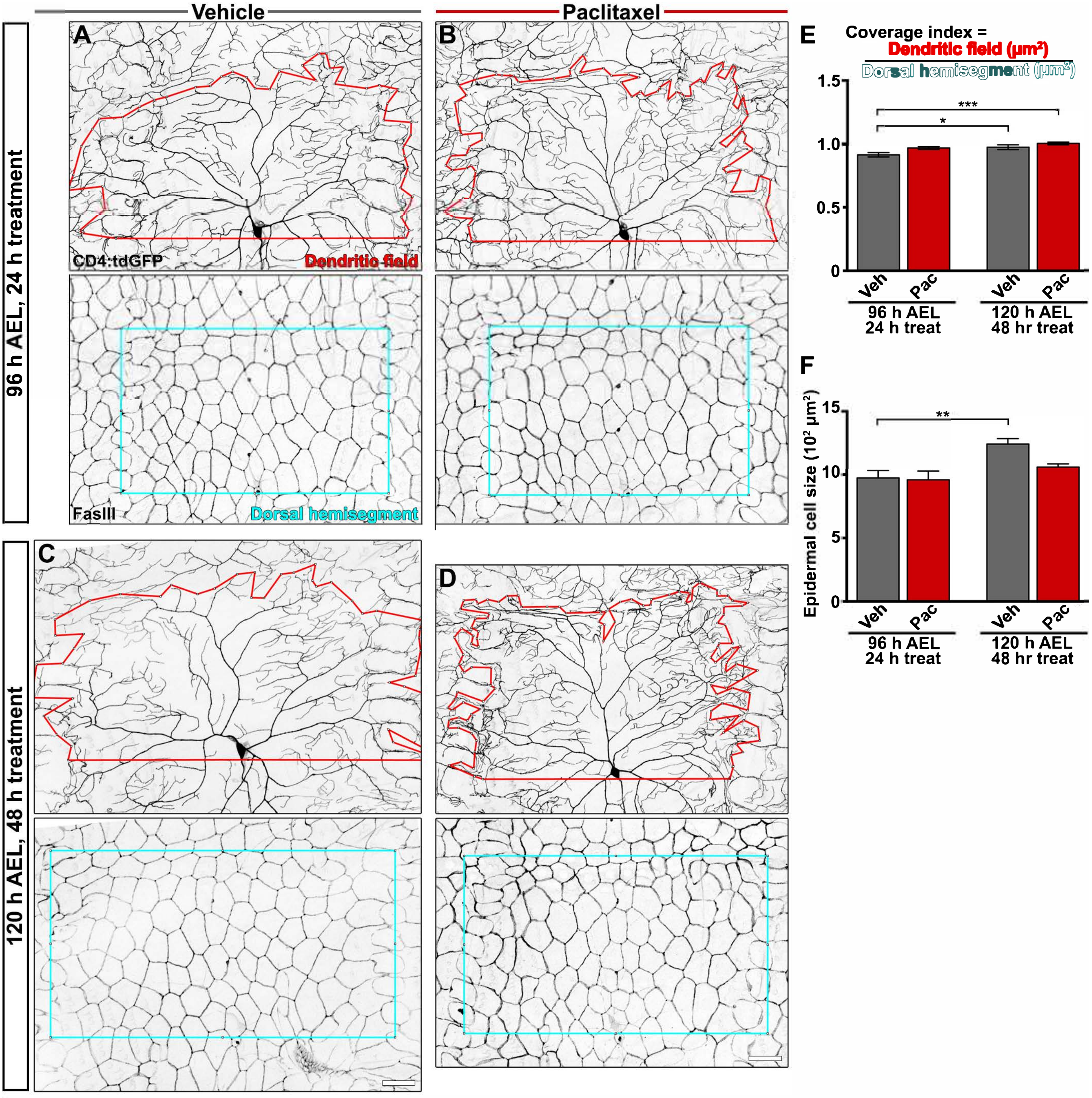

Figure S1. Paclitaxel does not alter scaling growth of dendrites during larval development

(A-D) Confocal projections of CD4:tdGFP labeled C4da neurons from vehicle- (A,C) and paclitaxel-treated (B,D) larvae and immunostaining for FasIII to label the epidermal cell boundaries. Red lines delineate the dendritic field covered by the dorsal projections of a single C4da neuron. Cyan lines indicate the area of the dorsal hemisegment containing the corresponding C4da dorsal dendrites. Scale bar, 50 µm

(E) The coverage index is defined as the area of the dendritic field divided by the dorsal hemisegment as in (Parrish et al., 2009). The coverage index approaches 1.0 as larvae develop from 96 h AEL to 120 h AEL. mean ± s.e.m.; n > 7 neurons from > 5 larvae form each time point and treatment; one-way ANOVA with Tukey's multiple comparisons test

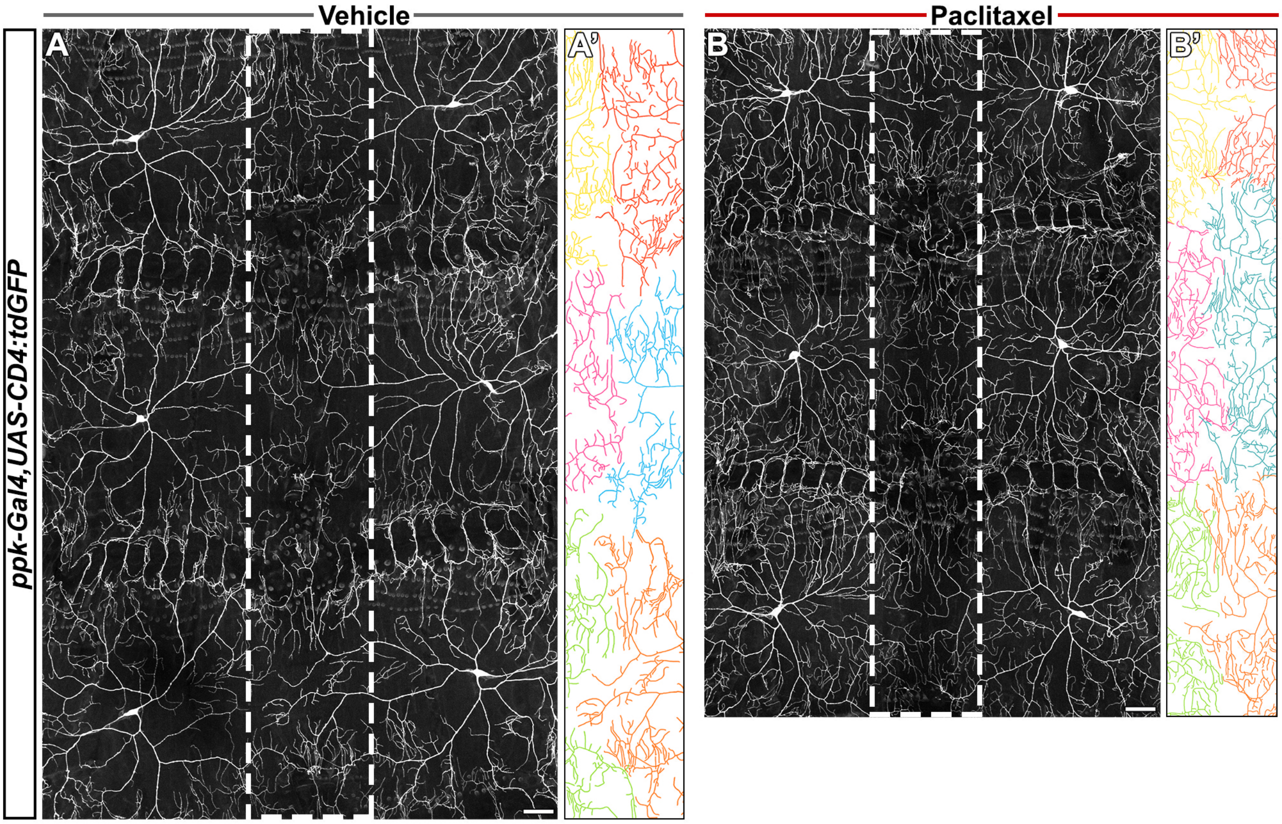

Figure S2. Paclitaxel does not disturb dendrite patterning

(A-B) Confocal projections of abdominal segments 3-5 from a vehicle- (A) and paclitaxel-treated (B) larva with CD4:tdGFP labeled C4da neurons. Dashed box designates region where dendrites from adjacent neurons approach one another and their hemisegment counterparts at the midline. (A'-B') Dendrites from individual neurons (indicated by dashed box in A-B) are pseudocolored to emphasize complete and nonredundant innervation characteristic of C4da dendrites. Upon paclitaxel treatment, C4da dendrites exhibit occasional crossover events (asterisks) with dendrites from neighboring neurons (B'). Scale bar, 50 µm

Disease Models & Mechanisms • Supplementary information

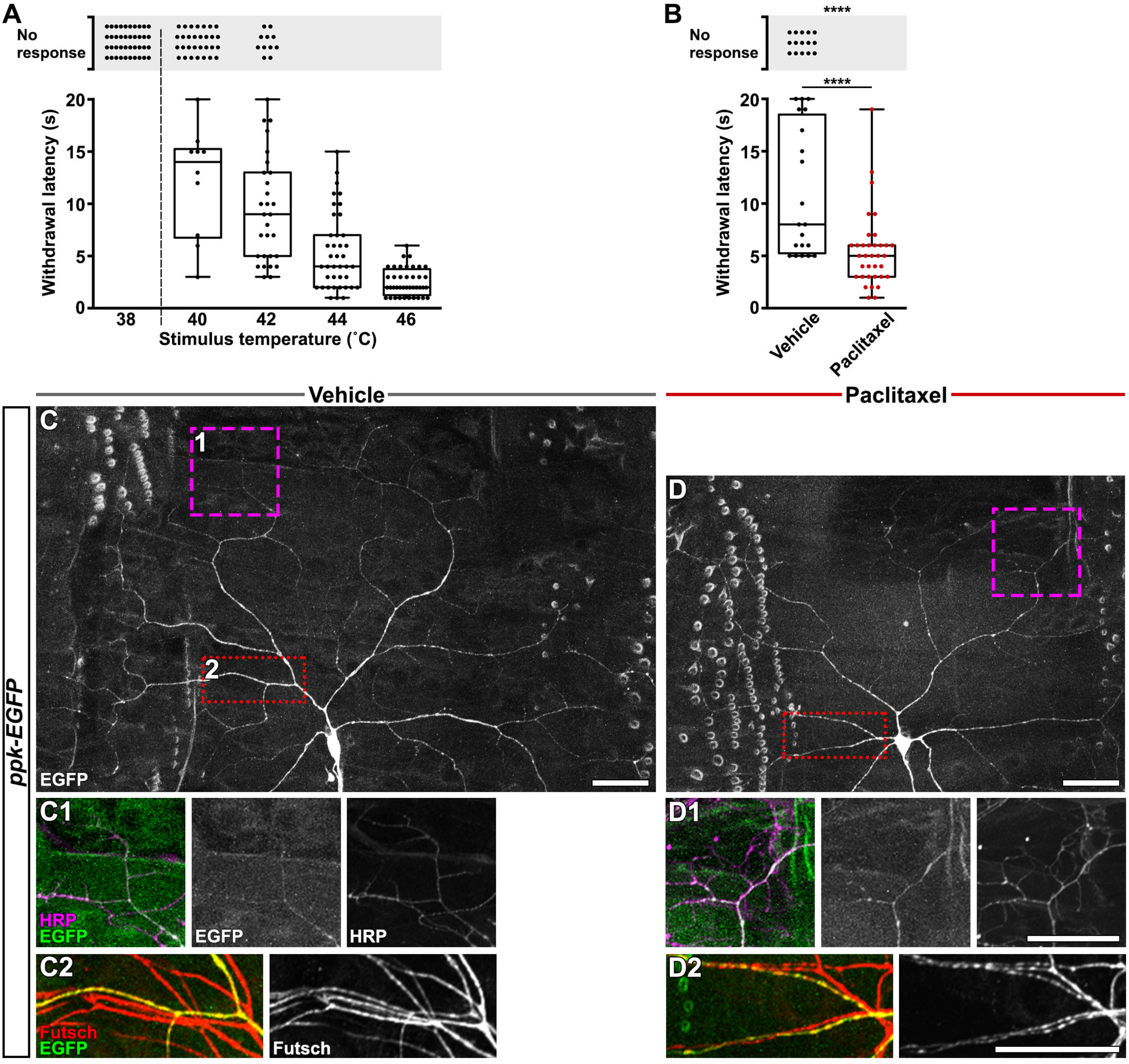

Disease Models & Mechanisms • Supplementary information

Figure S3. Paclitaxel induces cellular and behavioral phenotypes independent of fluorescent reporter fidelity

(A) Thermal nociceptive profile of third instar larvae expressing a cytosolic fluorescent reporter (*ppk-EGFP*) stimulated at indicated temperature. Each data point represents an individual larva scored for withdrawal latency (box and whiskers) or categorized as no response if aversive withdrawal was not initiated within 20 s of stimulation. No larvae responded at 38 ˚C indicating sub-threshold stimulation (dashed line). n = 40 larvae tested at each temperature

(B) Withdrawal response from 42 ˚C stimulation of late third instar (120 h AEL) larvae expressing *ppk-EGFP* after 48 h exposure to either vehicle or paclitaxel. n = 35 larvae for each treatment; withdrawal latency, Student *t* test; response rate, Mann-Whitney test

(C-D) Confocal projections of fixed, filet-dissected vehicle- or paclitaxel-treated larvae showing ddaC dendrites labeled by C4da specific expression of the cytosolic EGFP reporter. Dashed regions shown in high magnification with immunostaining in D1-E2. HRP immunostaining of neuronal membranes reveals fine terminal dendrites of C4da sensory neurons where cytosolic EGFP is absent (C1). Paclitaxel treated larvae exhibit increased HRP-labeled dendrites (D1). Major dendritic branches are characterized by continuous pattern of Futsch immunostaining in vehicle-treated larvae (C2), but a disorganized and clustered pattern in paclitaxel-treated larvae (D2).

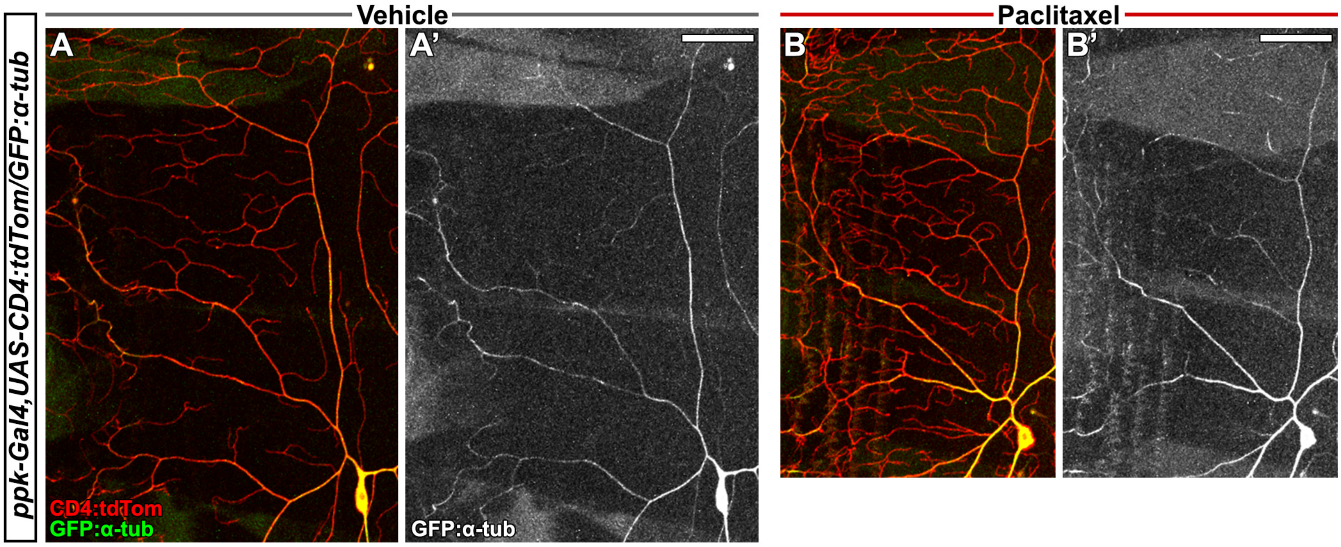

Figure S4. GFP:α-tubulin reporter cannot resolve paclitaxel-induced microtubule alterations

(A-B) Confocal projections from live imaging of genetically expressed GFP:α-tubulin within C4da ddaC sensory dendrites labeled with membrane-targeted CD4:tdTom from vehicle- (A) and paclitaxel-treated (B) larvae. Scale bar, 50 µm

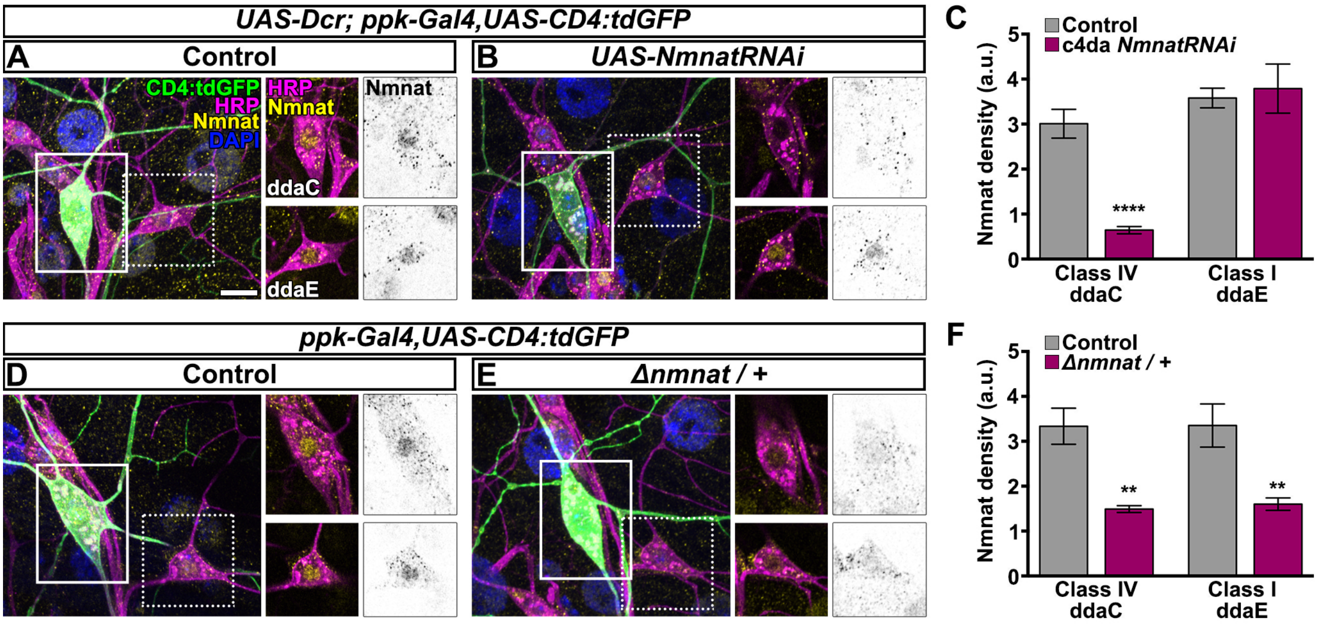

Figure S5. Levels of Nmnat protein by RNAi and copy number reduction approaches

(A-B) Confocal projections showing an overview of immunostaining of Nmnat within the peripheral tissue of larvae with C4da neuronal RNAi-mediated knockdown of Nmnat (B, *ppk-Gal4>UAS-Dcr,UAS-NmnatRNAi*) and controls (A, *ppk-Gal4>UAS-Dcr*). All peripheral neuronal membranes are stained with HRP, C4da neurons are labeled by CD4:tdGFP, and DAPI marks the nuclei of neurons as well as the surrounding muscle and epidermal cells. Solid boxed regions indicate Class IV ddaC nociceptors and dashed box regions indicate mechanosensitive Class III ddaE sensory neurons. Single z slices of ddaC and ddaE neurons are shown to resolve Nmnat localization and levels within the tissue of interest. Scale bar, 50 µm

(C) Quantification of Nmnat protein density from single z planes through the peripheral neuronal nuclei of Class IV ddaC and Class I ddaE neurons from control larvae and larvae with C4da RNAi-mediated Nmnat knockdown. mean ± s.e.m.; n = 5 neurons from > 4 larvae from each genotype, Student *t* test

(D-E) Confocal projections showing an overview of peripheral tissue of control larvae and larvae heterozygous for a *Nmnat* loss-of-function allele (*Δnmnat* / +). Solid boxed regions indicate Class IV ddaC neurons and dashed box regions indicate Class III ddaE neurons. Single z slices of ddaC and ddaE neurons are shown to resolve Nmnat within the tissue of interest. Scale bar, 50 µm

(F) Quantification of Nmnat protein density within peripheral neuronal nuclei of Class IV ddaC and Class I ddaE neurons from control larvae and *Δnmnat* heterozygous larvae. mean ± s.e.m.; n = 5 neurons from > 4 larvae from each genotype, Student *t* test
